# Cardiovascular adverse events of antineoplastic monoclonal antibodies among cancer patients: real-world evidence from a tertiary healthcare system

**DOI:** 10.1186/s40959-023-00184-z

**Published:** 2023-09-25

**Authors:** Abdulrazaq S. Al-Jazairi, Nahlah Bahammam, Dhai Aljuaid, Lama Almutairi, Shroog Alshahrani, Norah Albuhairan, Peter M. B. Cahusac, Ghazwa B. Korayem

**Affiliations:** 1 Division of Clinical Trials Transformation Initiative, King Faisal Specialist Hospital & Research Centre, PO Box 3354, Riyadh, 11211 Kingdom of Saudi Arabia; 2 College of Pharmacy and Medicine, Alfaisal University, P.O. Box 50927, Riyadh, 11533 Kingdom of Saudi Arabia; 3 College of Pharmacy, Princess Nourah Bint Abdulrahman University, P.O. Box 101283, 11655 Riyadh, Saudi Arabia; 4 King Faisal Specialist Hospital & Research Centre, PO Box 3354, Riyadh, 11211 Kingdom of Saudi Arabia; 5 Department of Pharmacy Practice, College of Pharmacy, Princess Nourah Bint Abdulrahman University, P.O. Box 84428, 11671 Riyadh, Saudi Arabia

**Keywords:** Monoclonal antibodies, Cardiovascular disease, Trastuzumab, Bevacizumab, Pertuzumab, Cardiovascular adverse event

## Abstract

**Background:**

Antineoplastic monoclonal antibodies (mAbs), such as trastuzumab, bevacizumab, and pertuzumab have been the mainstay of therapy in cancer patients. Despite proven efficacy of the monoclonal antibodies, cardiovascular-induced adverse events such as heart failure, hypertension, ischemic heart disease, arrhythmias, thromboembolic events, and hemorrhage remain a major complication. The European society of cardiology address that concern with antineoplastic monoclonal antibodies issuing a guideline to manage and monitor chemotherapy-induced cardiotoxicity. There is limited evidence of the real-world prevalence of cardiovascular (CV) events induced by monoclonal antibodies among patients with cancer in Saudi Arabia.

**Objective:**

To evaluate the prevalence of cardiovascular adverse events among patients with cancer treated with monoclonal antibodies in Saudi Arabia.

**Methods:**

This is a retrospective study conducted in a tertiary care hospital, Riyadh, Saudi Arabia. Data were obtained from an electronic medical record of patients with cancer treated with one of the selected monoclonal antibodies, who met the inclusion criteria between January 2005 until June 2015 and have been followed up for at least one year. Patients were stratified into groups according to monoclonal antibodies treatment: trastuzumab, bevacizumab, pertuzumab, and combined mAbs.

**Results:**

A total of 1067 patient were included in the study, within the pre-determined study period. The prevalence of cardiovascular disease among patients with cancer treated with monoclonal antibodies was 16.3%. The prevalence of heart failure was relatively higher in the trastuzumab group (46/626 patients, 7.3%). Among 418 patients treated with bevacizumab, hypertension was the most frequent adverse event, reported in 38 patients (9.1%), followed by thromboembolism reported in 27 patients (6.5%). Treatment discontinuation owing to cardiovascular adverse events was reported in 42/1,067 patients (3.9%).

**Conclusion and relevance:**

Prevalence of antineoplastic monoclonal antibody induced cardiovascular adverse events among patients with cancer is substantially high in Saudi Arabia. There is an urgent need to streamline the practice for identifying high risk patients and flexible referral system for cardio-oncology care.

## Introduction

Treatment with antineoplastic monoclonal antibodies (mAbs), which are anticancer agents, is associated with increased survival in patients with cancer [1]. Antineoplastic mAbs used in cancer therapy include trastuzumab, bevacizumab, and pertuzumab. Although these agents have beneficial effect on progression-free survival and overall survival when combined with other chemotherapy, they are still toxic at certain conditions [2–4]. Therefore, the United States Food and Drug Administration (FDA) has issued warnings regarding the risk of cardiomyopathies with mAb treatment, including heart failure, left ventricular dysfunction, in addition to hypertension, and hemorrhage [5–7].

The efficacy of bevacizumab as an adjuvant therapy in patients with metastatic colorectal cancer has been well established. Bevacizumab treatment is associated with improvements in progression-free and overall survival [8]. An evidence review has also proven the efficacy of trastuzumab treatment in women with human epidermal growth factor receptor 2-positive metastatic breast cancer, extending the progression-free and overall survival [9]. Moreover, pertuzumab has been proven to be a safe and effective drug for treating patients with solid tumors [10].

Despite the proven efficacy of antineoplastic mAbs in treating cancer, cardiovascular (CV) events associated with mAb treatment may be serious and can affect the patient’s quality of life and overall survival [4]. Previous studies have shown that the prevalence of bevacizumab-related CV events is 1.7–4% for heart failure [11–13], up to 36% for hypertension [14, 15], 3.8–10.9% for thromboembolism [16, 17], 1% for ischemic heart disease [18], and 5.8% for all hemorrhage events [19]. Whereas, the reported prevalence of trastuzumab-induced cardiac event was 11.3% out of 4,017 patients in a pooled analysis study [20], 1.2% for arrhythmia [21], 4% for hypertension, and 2% epistaxis [6]. The incidence of pertuzumab-induced cardiotoxicity has been reported to range from 3.4–6.5% for heart failure [12, 22] and 29% for hypertension [23].

Due to the emerging evidence on the development of CV events associated with antineoplastic mAb treatment, the European society of cardiology issued a guideline that addresses the CV events associated with chemotherapy and provides a guideline to manage and monitor chemotherapy-induced cardiotoxicity [24]. Therefore, there is a need to determine the real-world prevalence of CV events in patients using mAbs, particularly in relation to our patients and healthcare system in Saudi Arabia. Since a high prevalence of CV risk factors has already been reported among the Saudi population and poor overall control of these risk factors [25], this study was conducted to evaluate the prevalence of CV events associated with mAb treatment among patients in our governmental healthcare system and management of these adverse events in the practice.

## Materials and methods

### Study design

This retrospective study was conducted in a tertiary care setting, at King Faisal Specialist Hospital & Research Center, Riyadh, Saudi Arabia, with 300-bed oncology center. All patients who were treated with selected antineoplastic mAbs, including trastuzumab (Herzuma: IV Injection in vial: 440 mg), bevacizumab (Avastin®: Injection, solution: 25 mg/mL), and pertuzumab (Perjeta® Solution, injection: 420 mg/14 mL). In case of developing cardiovascular event for patients on combination of mAbs (two or more), the adverse event will be designated for the culprit combination under the results section. Patients using one or more of the selected mAb from January 2005 to June 2015 were included, to allow time for the five-year overall survival assessment. The inclusion criteria were as follows: age ≥ 18 years, diagnosis of cancer, and treatment with any of the three selected mAbs. Furthermore, patients must have been followed up for at least one year. Exclusion criteria included pediatric patients aged < 18 years, diagnosed with cancer and not treated with any of the three selected antineoplastic mAbs, and those who did not complete at least one-year follow-up duration. This study was conducted in compliance with the requirements of the Institutional Review Board/Human Subjects Research Committee and approved by our Institutional Research Advisory Council (RAC number 2191175).

### Data collection

All the data were collected from the electronic health records of the organization. The retrieved data included patient characteristics, medical history, and chemotherapy protocol. In addition to data on the development of CV events or worsening of a CV disease (CVD), data on the management of CV events, mAb dose adjustments based on CVD, hospital admission related to CVD, emergency department visits related to CVD, referral to cardiology clinic, and overall survival were collected. The data were collected manually utilizing a standard data collection form with clear definitions of all parameters and then entered in secure electronic software tool, REDCap™ software, version 6.3.0 – 2017, Vanderbilt University (Nashville, TN, USA).

### Outcomes

The primary outcome was the prevalence of CV events, particularly heart failure, hypertension, ischemic heart disease, arrhythmias, thromboembolism, and hemorrhage, associated with the use of antineoplastic mAbs within one year of therapy initiation. For patients who developed more than one CV event, each event was considered one encounter.

In this study, heart failure was reported as it was documented in the records of the patients, which was coded as heart failure with preserved ejection fraction, heart failure with reduced ejection fraction, congestive heart failure, decompensated heart failure, left ventricular dysfunction, or when the documented ejection fraction was < 40% [26]. Hypertension was defined according to the physician documentation or based on a newly prescribed antihypertensive agent. Ischemic heart disease was defined according to the physician documentation as either coronary artery disease, coronary heart disease, stable angina, non-ST segment elevation acute coronary syndrome, or ST-segment elevation acute coronary syndrome. Arrhythmia was defined according to the physician documentation of arrhythmia in patient records as sinus tachycardia, bradyarrhythmia, tachyarrhythmias, ventricular arrhythmia or supraventricular arrhythmia, QT prolongation, torsade de point, atrial fibrillation, and conduction defect. Thromboembolism was defined according to the physician documentation of thromboembolism in patient records as arterial thromboembolism, venous thromboembolism, coronary artery disease, cerebral artery ischemia, stroke, arterial embolism deep vein thrombosis, and pulmonary embolism. Hemorrhage was defined according to the physician documentation of bleeding in patient records: documentation of major bleeding, either mentioned as a decrease in hemoglobin level of at least 2 g/dl, requiring transfusion of at least two units, requiring surgical correction, or requiring intravenous vasoactive agents; minor bleeding that was mentioned as epistaxis, gastrointestinal bleeding, and vaginal bleeding [27]. This included worsening of heart failure, hypertension, ischemic heart disease, or arrhythmia based on increased dose or additional medication.

Secondary outcomes included use of medication to manage mAb-induced CV events; mAb dose adjustments, or discontinuation due to CV events; CV event-related hospital admissions; CV event-related emergency department visits; number of patients referred to the cardiovascular clinic; and overall survival. A probability scaling was used to assess the temporal event-agent relationship [28].

### Statistical analysis

Chi-squared tests of association were performed on categorical data. Survival analysis was performed on overall survival data for different treatments. Comparisons of means were performed using Welch’s t-test. The critical probability for statistical significance was 0.05. Statistical analyses were performed using jamovi (jamovi project (2022), Version 2.3 [Computer Software], Sydney, Australia; retrieved from https://www.jamovi.org, August 2, 2023).

## Results

**Fig. 1:**
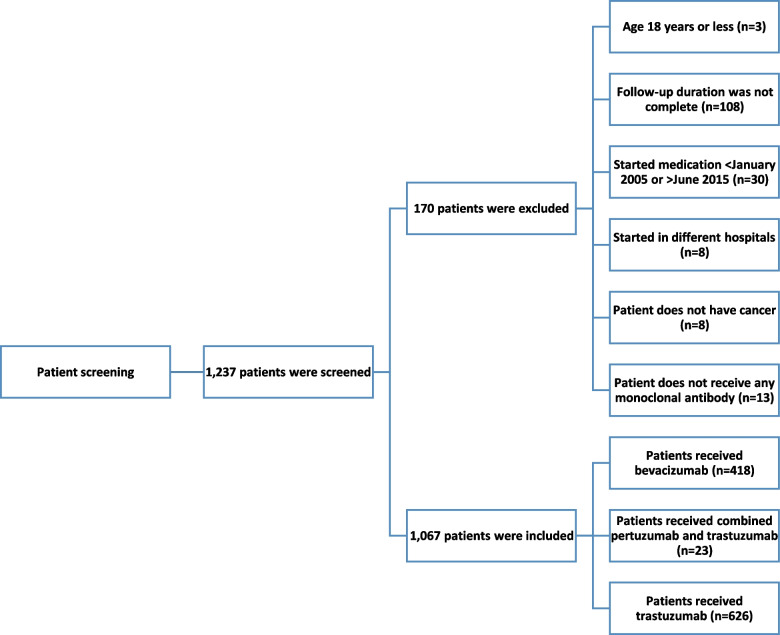
Screening of patients with cancer according to exclusion and inclusion criteria

Out of the 1,237 screened patients, 1,067 satisfied the selection criteria and were included in this study (Fig. 1). Most of the patients were treated with trastuzumab (*n* = 626, 58.7%), followed by bevacizumab (418, 39.2%), and the rest were treated with combined pertuzumab and trastuzumab (23, 2.1%). The majority of the patients were women (78.7%), with a mean age of 41 ± 11.7 years (Table 1). Baseline blood pressure, ejection fraction, and related laboratory results are summarized in Table 2. The most common indication for trastuzumab and pertuzumab treatments was breast cancer, whereas bevacizumab was mostly used for treating colorectal cancer.

**Table 1:** Baseline characteristics of the patients with cancer who received antineoplastic mAbs (*N* = 1,067)

Characteristics	Patients on antineoplastic mAbs (*n* = 1067)				
**Monoclonal Antibody**	**Total** ***N* = 1067**	**Trastuzumab** ***n* = 626**	**Bevacizumab** ***n* = 418**	**Pertuzumab** **combined with trastuzumab** ***n* = 23**	***p*-value**
**Age at mAb initiation in years, Mean ± SD**	49 ± 11.77	46.4 ± 10.7	53.2 ± 12.1	42.1 ± 9.9	< 0.001
**Female, n (%)**	840 (78.7%)	617 (98.6%)	200 (48%)	23 (100%)	< 0.001
**Body mass index (kg/m**^**2**^**), Mean ± SD**	29 ± 6.24	30.6 ± 6.1	26.7 ± 5.7	28.8 ± 5	< 0.001
	**Comorbidities**, **n (%)**				
**Diabetes mellitus**	237 (22.2%)	122 (19.4%)	113 (27%)	2 (8.7%)	0.004
**Dyslipidemia**	68 (6.4%)	42 (6.7%)	26 (6.2%)	0 (0%)	0.427
**Anemia**	14 (1.3%)	5 (0.8%)	8 (1.9%)	0 (0%)	0.240
**Hyperthyroidism**	7 (0.7%)	4 (0.63%)	3 (0.7%)	0 (0%)	0.914
**Hypothyroidism**	66 (6.2%)	43 (6.9%)	20 (4.8%)	3 (13%)	0.151
**Hypertension**	241 (22.6%)	114 (18.2%)	124 (29.7%)	3 (13%)	< 0.001
**Heart failure**	4 (0.4%)	3 (0.48%)	1 (0.24%)	0 (0%)	0.789
**Ischemic heart disease**	15 (1.4%)	8 (1.3%)	7 (1.7%)	0 (0%)	0.734
**Arrhythmia**	10 (0.9%)	7 (1.1%)	3 (0.7%)	0 (0%)	0.721
	**mAb indication and duration of therapy**				
**Breast cancer, n (%)**	647 (60.6%)	608 (97.1%)	16 (3.8%)	23 (100%)	< .001
**Gastric cancer, n (%)**	16 (2.6%)	16 (2.6%)	0	0	0.003
**Endometrial cancer, n (%)**	2 (0.3%)	2 (0.3%)	0	0	0.494
**Colorectal cancer, n (%)**	359 (85.9%)	0	359 (85.9%)	0	< 0.001
**Ovarian cancer, n (%)**	11 (2.6%)	0	11 (2.6%)	0	< .001
**Glioblastoma, n (%)**	8 (1.9%)	0	8 (1.9%)	0	0.002
**Other, n (%)**	7 (1.7%)	0	7 (1.7%)	0	0.004
**Duration of therapy (months), mean**	14.4	16	11.8	12.8	< 0.001
	**mAb concomitant medication**				
**Docetaxel, n (%)**	474 (44.4%)	455 (72.7%)	6 (1.4)	13 (56.5%)	< 0.001
**Cyclophosphamide, n (%)**	68 (6.3%)	67 (10.7%)	1 (0.2%)	0	< 0.001
**Carboplatin, n (%)**	45 (4.2%)	38 (6%)	7 (1.7%)	0	0.001
**Paclitaxel, n (%)**	52 (4.8%)	35 (5.6%)	13 (3.1%)	4 (17.4%)	0.004
**Cisplatin, n (%)**	20 (1.87%)	18 (2.9%)	2 (0.5%)	0	0.016
**Capecitabine, n (%)**	90 (8.43%)	12 (2%)	78 (18.6%)	0	< 0.001
**Vinorelbine, n (%)**	6 (1%)	6 (1%)	0	0	0.119
**Fluorouracil, n (%)**	50 (4.6%)	6 (1%)	44 (10.5%)	0	< 0.001
**Doxorubicin, n (%)**	9 (0.84%)	3 (0.5%)	5 (1.2%)	1 (4.3%)	0.082
**Oxaliplatin, n (%)**	247 (23%)	3 (0.5%)	244 (58.4%)	0	< 0.001
**Lapatinib, n (%)**	2 (0.3%)	2 (0.3%)	0	0	0.494
**Gemcitabine, n (%)**	3 (0.28%)	1 (0.2%)	2 (0.5%)	0	0.614
**Epirubicin, n (%)**	1 (0.2%)	1 (0.2%)	0	0	0.703
**Irinotecan, n (%)**	134 (32%)	0	134 (32%)	0	< 0.001
**Leucovorin, n (%)**	39 (9.3%)	0	39 (9.3%)	0	< 0.001
**Pemetrexed, n (%)**	2 (0.5%)	0	2 (0.5%)	0	0.211
**Topotecan, n (%)**	2 (0.5%)	0	2 (0.5%)	0	0.211
**None, n (%)**	134 (12.5%)	93 (14.9%)	41 (9.8%)	0	0.010

*mAb* monoclonal antibody

**Table 2:** Baseline blood pressure, ejection fraction, and related laboratory results (*N* = 1,067)

	**Mean**	**Median**	**Standard deviation**
Systolic Blood pressure (mmHg)	127	125	13.82
Diastolic Blood pressure (mmHg)	77	78	9.39
Ejection fraction (%)	54	55	4.169
B-type natriuretic peptide level (pg/mL)	139	0	0
Troponin T (ng/mL)	1.5	43	0
Creatine kinase (U/L)	69.07	160	0
Calcium level (mmol/L)	2.28	2.25	0.138
Magnesium level (mmol/L)	0.8	0.84	8.92
Potassium level (mmol/L)	4.14	4.2	0.452
Sodium level (mmol/L)	139.76	140	3.72
Albumin (g/L)	39.85	39	5.89
Hemoglobin (g/L)	119.05	117	17.22
Hematocrit (L/L)	0.36	0.361	0.045
Serum creatinine (μmol/L)	62.38	60	16.64
GFR (mL/min/1.73 m2)	50.25	60	0.816
AST (U/L)	28.1	20	27.36
ALT (U/L)	26.42	17.05	26.23
Total Bilirubin (μmol/L)	7.02	6	4.87
INR	1.12	1	0.102
Prothrombin time (s)	30.40	34.35	4.76

*ALT* alanine transaminase, *AST* aspartate aminotransferase, *GFR* glomerular filtration rate, *INR* international normalized ratio

Collectively, the prevalence of CV events in patients receiving trastuzumab, bevacizumab, and pertuzumab was 16.3%. Heart failure was relatively more common among patients treated with trastuzumab (7.3%), followed by pertuzumab combined with trastuzumab (4.3%) and bevacizumab (0.7%), *p* < 0.0001. Hypertension was more frequently reported in patients treated with combined pertuzumab and trastuzumab (26.1%) than in those treated with bevacizumab (9.1%) or trastuzumab (2.6%), *p* < 0.001. Thromboembolism or hemorrhage was more frequently reported in patients treated with bevacizumab (10.8%) than in those treated with trastuzumab (1.9%) or combined pertuzumab and trastuzumab (4.3%), *p* < 0.0001, (Table 3). The probability of the temporal relationship between these CV events and the use of these three mAbs is summarized in Table 4.

**Table 3:** Prevalence of CV events among patients with cancer treated with the three antineoplastic monoclonal antibodies (*N* = 1,067)

CV Event	**Total** **(*N* = 1067)**	**Trastuzumab** **(*n* = 626)**	**Bevacizumab** **(*n* = 418)**	**Pertuzumab combined with trastuzumab** **(*n* = 23)**	***p-value***
Overall CV events, n (%)	**174 (16.3%)**	**77 (12.3%)**	**89 (21.2%)**	**8 (34.7%)**	< 0.001
Heart failure, n (%)	50 (4.7%)	46 (7.3%)	3 (0.7%)	1 (4.3%)	< 0.001
Hypertension, n (%)	60 (5.6%)	16 (2.6%)	38 (9.1%)	6 (26.1%)	< 0.001
Ischemic heart disease, n (%)	2 (0.19%)	1 (0.15%)	1 (0.24%)	0	0.940
Arrhythmias, n (%)	4 (0.37%)	2 (0.31%)	2 (0.47%)	0	0.880
Thromboembolism, n (%)	32 (3%)	4 (0.6%)	27 (6.5%)	1 (4.3%)	< 0.001
Hemorrhage, n (%)	26 (2.4%)	8 (1.3%)	18 (4.3%)	0	0.006

*CV* cardiovascular

**Table 4:** ADR probability scale for suspected ADRs associated with antineoplastic monoclonal antibodies [28]

	**Total Number**	**%**	**Doubtful**^**a**^ **ADRs**	**Possible**^**b**^ **ADRs**	**Probable**^**c**^ **ADRs**	**Definite**^**d**^ **ADRs**
Heart failure	50	4.7%	0	25	25	0
Hypertension	60	5.6%	1	46	13	0
Ischemic heart disease	2	0.19%	0	2	0	0
Arrhythmia	4	0.37%	0	3	1	0
Thromboembolic event	32	3.0%	1	22	8	1
Hemorrhage	26	2.4%	1	20	5	0

*ADR* adverse drug reaction

^a^ Naranjo scale score from 0 or lower

^b^ Naranjo scale scores from 1 to 4

^c^ Naranjo scale score from 5 to 8

^d^ Naranjo scale score ≥ 9

**Fig. 2:**
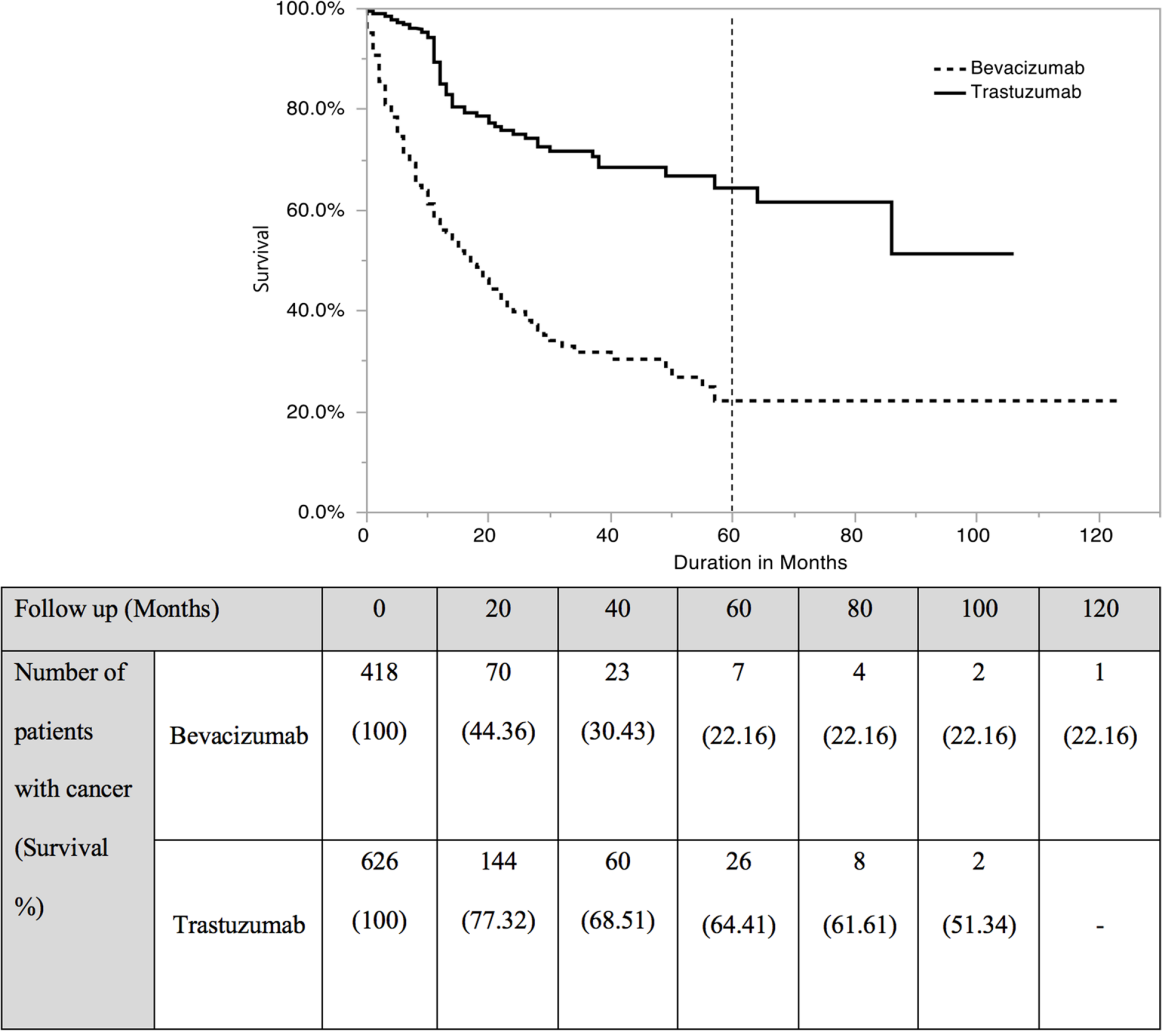
Overall survival of patients receiving trastuzumab and bevacizumab

Regardless of the treatment of the underlying cancer and its prognosis, the five-year survival associated with bevacizumab treatment and trastuzumab treatment were 22.1% and 64.4%, respectively; the overall survival is illustrated by a Kaplan–Meier plot. However, the median survival of patients receiving bevacizumab was 17 months (Fig. 2).

Antineoplastic mAb treatment was discontinued owing to development of CV events by trastuzumab in 42.2% of the cases and by bevacizumab in 14.8% of cases. None for those treated by combined pertuzumab and trastuzumab (these proportions were statistically significant, *p* < 0.001. Combined pertuzumab and trastuzumab treatment was not discontinued based on any CV event. Antineoplastic mAb treatment dose was adjusted in 9 of 174 patients (5.1%) who developed CV events. Angiotensin-converting enzyme inhibitors (ACE-Is) were the most commonly used agents for treating heart failure and hypertension induced by antineoplastic mAbs. The management of the different CV events varied depending on the event presented (Table 5A–F).

**Table 5:** Management of cardiovascular adverse event associated with monoclonal antibody

**A. HF management (*n*** **= 49)**				
		**Count**	**Total**	**%**
Was HF treated?	Yes	33	49	67.3%
ACE inhibitors	Yes	24	33	72.7%
ARBs	Yes	1	33	3%
Beta-blockers	Yes	20	33	60.6%
Diuretics	Yes	7	33	21.2%
Digoxin	Yes	2	33	6.1%
Other treatments	Hold trastuzumab	2	33	6.1%
**B. HTN management (*n*** **= 34)**				
		**Count**	**Total**	**%**
Was HTN treated?	Yes	34	34	100%
ACE inhibitor	Yes	18	34	52.9%
ARBs	Yes	1	34	2.9%
Diuretics	Yes	3	34	8.8%
CCBs	Yes	11	34	32.4%
Beta-blockers	Yes	9	34	26.5%
Other treatments	Spironolactone	1	34	2.9%
**C. IHD management (*n*** **= 2)**				
		**Count**	**Total**	**%**
Was IHD treated?	Yes	2	2	100%
ASA	Yes	1	2	50%
ACE inhibitors	Yes	2	2	100%
Beta-blockers	Yes	2	2	100%
Heparin	Yes	1	2	50%
Other treatments	Clopidogrel and Simvastatin	1	2	50%
**D. Arrhythmia management (*n*** **= 3)**				
		**Count**	**Total**	**%**
Was arrhythmia treated?	Yes	2	3	66.7%
Class II (beta-blockers)	Yes	2	2	100%
Class III (K channel-blockers)	Yes	1	2	50%
Class IV (Ca channel-blockers)	Yes	1	2	50%
Other treatments	Enoxaparin	1	2	50%
**E. Thromboembolism management (*n*** **= 32)**				
		**Count**	**Total**	**%**
Was thromboembolic event treated?	Yes	29	32	90.6%
Warfarin	Yes	2	29	6.9%
LMWH	Yes	27	29	93.1%
UFH	Yes	3	29	10.3%
Other treatments	Hold bevacizumab	2	29	6.9%
**F. Hemorrhage management (*n*** **= 25)**				
		**Count**	**Total**	**%**
Was hemorrhage treated?	Yes	10	25	40%
FFP	Yes	1	10	10%
Platelet concentrates	Yes	1	10	10%
Surgery	Yes	1	10	10%
Other treatments	Yes	7	10	70%

*ACE* angiotensin-converting enzyme, *ARB* angiotensin receptor-blocker, *ASA* aspirin, *CCB* calcium channel blocker, *FFB* fresh frozen plasma, *HF* heart failure, *HTN* hypertension, *IHD* ischemic heart disease, *LMWH* low-molecular-weight heparin, *UFH* unfractionated heparin

Patients receiving bevacizumab had more emergency visits due to CV event over one year (mean = 0.753, range 0 – 9) than those receiving trastuzumab (mean = 0.197, range 0 – 2), *p* < 0.001. The proportion of those making emergency visits who received bevacizumab was 34 of 81 (42%), while for those who received trastuzumab it was 10 of 71 (14%). The difference between these proportions was statistically significant, *p* < 0.001. No emergency department visits were made for patients receiving combined pertuzumab and trastuzumab.

Conversely, among patients who developed CV events the number of visits to the cardiology clinic over one year was higher for patients receiving trastuzumab (mean = 3.191), with a mean duration of therapy of 16 months than those who received bevacizumab (mean = 0.414) with a mean duration of therapy of 11.8 months. The difference in the mean number of visits between the two treatments was statistically significant, *p* < 0.001. The proportion of those making cardiology clinic visits who received trastuzumab was 41 of 47 (87%), while for those who received bevacizumab it was only 8 of 32 (25%). The difference in these proportions was also statistically significant, *p* < 0.001. Only one patient who received combined pertuzumab and trastuzumab visited the cardiology clinic.

Hospital admissions owing to CV events were reported more frequently for patients receiving bevacizumab (37 patients, 45.7%) than for patients receiving trastuzumab (16, 22.5%), with no hospital admission reported for patients receiving pertuzumab combined with trastuzumab, *p* = 0.002, (Table 6).

**Table 6:** Admissions and referral for patients who developed cardiovascular events in response to the antineoplastic monoclonal antibodies

Drug	Hospital admission	Emergency department visits	Cardiology clinic referral
Bevacizumab (*n* = 81 patients)	37 (45.7%)	61 (75.3%)	8 (9.8%)
Trastuzumab (*n* = 71 patients)	16 (22.5%)	14 (19.7%)	41 (57.7%)
Pertuzumab combined with trastuzumab (*n* = 6 patients)	0	0	1 (16.7%)
Total (*n* = 158 patients)	53 (33.5%)	75 (47.4%)	50 (31.6%)

## Discussion

Cardiovascular adverse events represent a major concern in the use of targeted therapies for patients with cancer. The association of these drugs with such events affects the quality of life and overall survival of patients [29, 30]. As the number of patients treated with biological drugs is continuously increasing, the incidence of cardiotoxicity is also increasing [31, 32]. In this study, the overall CV events associated with antineoplastic mAb treatment were reported once in every six patients (16.3%). This highlights the importance of building infrastructure to improve the screening, diagnostic, and management burdens of these events, preferably by establishing or expanding cardio-oncology clinics [33]. The FDA approved the use of a serial cardiac evaluation that should be implemented every 3 months throughout trastuzumab treatment [34]. In contrast, The European Society for Medical Oncology has issued a guideline stating that left ventricular ejection fraction assessment should be performed at least every 3 months during trastuzumab treatment [35].

Heart failure was the most common CV event among patients treated with trastuzumab, with an incidence rate of 7.3%. Notably, this finding is consistent with the previously reported incidence rate of 7.4% [36]. There are infrequent reports of heart failure incidence in patients receiving bevacizumab of approximately 4% and as high as 14% when used in a combination therapy [37].

Docetaxel was frequently used as a concomitant therapeutic regimen; however, it was associated with low cardiotoxicity risk [38]. The management of heart failure should include an overall CV risk assessment and individual clinical evaluation. In our study, most patients were treated with ACE-Is and beta-blockers, which have been reported to show good results [34].

Hypertension was reported more frequently with bevacizumab treatment (9%, 38 patients), which still falls within the reported incidence range of 4–35% [39]. For bevacizumab-induced hypertension, clinical trials do not recommend any specific antihypertensive treatment and the treatment is provided based on the physician's discretion [40]. Certain studies recommend the use of aggressive treatment with ACE-Is or dihydropyridine calcium channel-blockers (CCBs) [41, 42]. In this study, most patients were found to be treated with ACE-Is or CCB for bevacizumab-induced hypertension. Hypertension was reported in combined pertuzumab and trastuzumab group by (26%, 6 patients) which was also presented in a previous study by 20 patients (5.5%) [43].

Thromboembolism was reported mainly in the bevacizumab group with an incidence rate of 6.5%. In a systematic review of 22 randomized controlled trials that included 13,185 patients treated with bevacizumab, the incidence rate was reported as 9.9% compared with 7.5% in the control group [44]. In this study, thromboembolic events were mostly managed using low-molecular-weight heparin, although data on the efficacy of new oral anticoagulants are emerging.

Bleeding is another important complication of bevacizumab therapy. Low-grade hemorrhage was the most common type of bleeding adverse event [45]. Among patients treated with bevacizumab; the reported overall incidence of hemorrhage was 4.3% in the present study compared to 5.8% in previous randomized controlled trial. Low-grade hemorrhage does not require any specific treatment [19]. In the present study, the majority of these cases were of low-grade severity, while 40% of patients who experienced hemorrhage required an intervention; 10% of cases were managed using fresh frozen plasma, 10% were managed using platelet concentrates, and 10% needed surgical intervention.

In a meta-analysis of 15 studies that included 8,124 patients to assess the risk of arrhythmia in patients with breast cancer treated with trastuzumab, the incidence of arrhythmia has been found to be 1.2% [21]. The rate reported in this meta-analysis is higher than that found in this study (0.31%).

Ischemic heart disease was observed in only two patients: one in the bevacizumab group and another in the trastuzumab group. However, bevacizumab has been associated with an increased risk of developing cardiac ischemia [46]. It is mainly managed using ACE-Is and beta-blockers.

Interestingly, patients receiving trastuzumab had a longer survival rate than that of those receiving bevacizumab, which is most likely related to the prognosis of the different types of cancers that the patients had, rather than the medication itself. As stated by the National Cancer Institute, the five-year relative survival rates for colorectal cancer and breast cancer from 2011 to 2017 were 64.7% and 90.3%, respectively [47, 48]. Both rates are higher than the rate reported in our study. A study demonstrated that adding bevacizumab to the treatment regimen for patients with metastatic colorectal cancer increases the median overall survival from 15.6 months to 20.3 months which is relatively higher than what was found in this study [49]. In contrast, other studies reported that bevacizumab treatment for ovarian cancer has no significant advantage in improving the overall survival of 16.6 months [50]. Despite bevacizumab is known to have more CV events, this did not associate with more cardio-oncology visits. This can be explained by the fact that cardio-oncology clinics are a newly established service and some of the prescribers of bevacizumab feel comfortable managing its CV adverse events.

This is the first and possibly the largest study to present real-world data on the prevalence of CV adverse events induced by antineoplastic mAbs in Saudi Arabia. However, this study had certain limitations. The retrospective design had shortcomings, including poor documentation, and loss of follow-up.

Quantifying and characterizing the prevalence and seriousness of CV adverse events associated with the use of antineoplastic mAbs is paramount. It has a significant impact on the healthcare system and requires meticulous CV screening, resilient referral systems, diagnosis, management, and monitoring. This monitoring can be implemented via a specialized cardio-oncology multidisciplinary service. The benefits of establishing a cardio-oncology clinic include the early detection of cancer therapy-related CV toxicity, facilitation of diagnosis, assessment of patient risk for CV complications at baseline before the initiation of cancer treatment, management of CV events, and long-term follow-up to help patients with CVD. Better communication between cardiologists and oncologists improves decision making, leading to better treatment and enhanced patient care [51].

## Conclusion and relevance

The use of antineoplastic mAbs was shown to substantially improve survival in patients with cancer. However, it also increases the risk of serious CV events. The prevalence of CV events in this study was considerably high. Therefore, practitioners should closely monitor these side effects at baseline and on a regular basis. Establishing specialized multidisciplinary services, such as cardio-oncology services, may help improve patient monitoring and management of CV events. Moreover, this study addresses this serious concern and encourages more research in the field.

## Data Availability

The data that support the findings of this study are available from the corresponding author upon reasonable request.

## Abbreviation

mAbs: Monoclonal antibodies
CVD: Cardiovascular disease
CV: Cardiovascular
FDA: U.S. Food and Drug Administration
